# Residents underestimate their robotic performance: evaluating resident robotic console participation time

**DOI:** 10.1007/s11701-023-01790-w

**Published:** 2024-05-10

**Authors:** Lauren Yoder, Nora Elson, Angela N Fellner, Katherine Meister, Hamza Guend

**Affiliations:** 1 TriHealth Surgical Institute, 375 Dixmyth Ave, Cincinnati, OH 45220 USA; 2 TriHealth Hatton Research Institute, Cincinnati, Ohio USA

**Keywords:** Robotic surgery, General surgery, Resident education, Autonomy

## Abstract

Lack of formal national robotic curriculum results in a void of knowledge regarding appropriate progression of autonomy in robotic general surgery training. One midwestern academic surgical training program has demonstrated that residents expect to independently operate more on the robotic console than they perceive themselves to do. As such, our study sought to evaluate expectations of residents and faculty regarding resident participation versus actual console participation time (CPT) at a community general surgery training program. We surveyed residents and faculty in two phases. Initially, participants were asked to reflect on their perceptions and expectations from the previous six months. The second phase included surveys (collected over six months) after individual cases with subjective estimation of participation versus CPT calculated by the Intuitive Surgical, Inc. MyIntuitive application. Using Mann–Whitney *U*-Test, we compared resident perceptions of CPT to actual CPT by case complexity and post-graduate year (PGY). Faculty (*n* = 7) estimated they allowed residents to complete a median of 26–50% of simple and 0–25% of complex cases in the six months prior to the study. They expected senior residents (PGY-4 and PGY-5) to complete more: 51–75% of simple and 26–50% of complex cases. Residents (*n* = 13), PGY-2–PGY-5, estimated they completed less than faculty perceived (0–25% of simple and 0–25% of complex cases). Sixty-six post-case (after partial colectomy, abdominoperoneal resection, low anterior resection, cholecystectomy, inguinal/ventral hernia repair, and others) surveys were completed. Residents estimated after any case that they had completed 26–50% of the case. However, once examining their MyIntuitive report, they actually completed 51–75% of the case (median). Residents, especially PGY-4 and 5, completed a higher percentage than estimated of robotic cases. Our study confirms that residents can and should complete more of (and increasingly complex) robotic cases throughout training, like the transition of autonomy in open and laparoscopic surgery.

## Background

Over the past decade, robotic surgery has developed an ever-increasing role in general surgery practice. Thus, training residents on use of the robotic platform has become a new challenge within the general surgery residency curriculum. In a recent study, 92% of surgery programs polled indicated that residents are involved in robotic surgeries and 84% of these programs indicated that residents are operating on the robotic console [1]. The majority of cases that residents participated in were hernia/soft tissue cases, closely followed by colorectal and biliary cases. There was no significant difference between residencies associated with a university and independent/community programs.

Despite initial efforts of the Fundamentals of Robotic Surgery consortium in 2014 [2], no standardized or required robotic curriculum for residents exists [3]. In the same study as above, only 68% of programs polled had a formal robotic surgery curriculum [1]. Requirements of these curricula vary widely. Most include a mix of robotic simulator modules, case observation, online courses (may or may not be industry sponsored), and live animal and cadaver labs [4]. Many of these protocols are also adapted from industry sponsored curricula developed for more experienced attending surgeons [5]. Additionally, residents may or may not actually complete these elements prior to actively participating in robotic surgery cases [6].

Certainly, no curriculum exists which dictates the progression of responsibility and autonomy of residents on robotic platforms during live operations as they become more familiar with the platform [5]. In all disciplines, residents and faculty members often have varied perceptions of the appropriate amount of resident autonomy in the operating room [7, 8]. With the introduction of robotic technology, this is no different. The Ohio State University Center for Minimally Invasive Surgery examined the difference between trainee expectations and reality of console participation time per case for robotic cholecystectomies and inguinal hernia repairs in 2019 [9]. The Ohio State group found that their robotic trainees expect to do more on the console than they perceive themselves to be completing.

In this study, we seek to understand the perceptions and expectations of residents and faculty at a community-based general surgery training program regarding resident participation on the robotic console on various case types. We compared this to the true amount of participation as measured by console participation time (CPT). We also investigated if these perceptions, expectations, and CPT vary by post-graduate year (PGY) and simple and complex case type to determine if this could guide curriculum development at our program.

## Methods

Prior to beginning this study, faculty were asked about their perceptions and expectations regarding resident participation in robotic cases (survey included in Appendix A). This pre-study survey was completed by faculty in December of 2021. They were asked to estimate in the last 6 months the average percent of simple and complex robotic cases they allowed residents to complete independently. Inguinal hernia repair, cholecystectomy, and partial colectomy were considered simple cases. Complex cases included low anterior resection (LAR), abdominoperineal resection (APR), pancreaticoduodenectomy, hepatectomy, abdominal wall reconstruction/midline hernia repair, and esophagectomy. “Independently” was defined as resident controlling the console but faculty giving verbal coaching. Faculty were then asked to describe the percent of simple and complex cases they expected residents at junior (PGY-2 and PGY-3) and senior (PGY-4 and PGY-5) levels to be able to complete.

Residents were asked also during December of 2021 to complete a similar pre-study survey (included in Appendix B). Residents were asked their PGY level and if they had completed a robotic surgery curriculum created by their residency program. They were then asked to estimate the average percent of simple and robotic cases they had completed in the last six months. They were also asked to report the percentage of simple and complex cases they expected a resident at their PGY level to complete.

Once the study was initiated, all residents created a profile within the MyIntuitive application created by Inituitive Surgical. Residents and faculty log in when sitting at the robotic console. After a robotic case is concluded, the software develops a report describing the instruments used, operative time for all surgeons involved, and procedure trends. For all cases completed from January to June 2022 with use of a teaching console (two consoles in the operating room), residents were encouraged to complete a post-case survey (included in Appendix C). The post-case survey indicated the resident’s academic year, case participated in, percent of time they perceived they completed, and percent of time actually completed (as determined by the MyIntuitive application). Residents were told to reflect on the time they perceived they completed prior to examining the MyIntuitive application case description (sample case included in Appendix C).

After six months of data collection, surveys were analyzed. Survey responses were compared between pre-case faculty expectations and resident expectations as well as post-case resident perceptions and actual case completion. Statistics were analyzed using Prism 8 (GraphPad Software, La Jolla, CA, USA). The categorical ordinal survey data were analyzed using Mann–Whitney U test. Figures were created using Microsoft Excel (Seattle, WA, USA) and Adobe Illustrator (Ventura, CA, USA).

## Results

### Faculty perceptions and expectations

Seven faculty members answered a survey assessing their perceptions and expectations surrounding resident involvement in robotic cases. On average (median), attending surgeons estimated that they allowed residents to complete 26–50% of simple cases and 0–25% of complex cases (see Table 1). When asked about their expectations, faculty expected PGY-2 and PGY-3 residents should be able to complete 26–50% of simple cases and 0–25% of complex cases. However, they expected more senior residents (PGY-4 and PGY-5) to complete 51–75% of a simple case and 26–50% of a complex case.

**Table 1:** Pre-study survey results

	Simple	Complex
Faculty pre-study survey		
Estimated allowed completion all levels	26–50%	0–25%
Expectations of junior residents	26–50%	0–25%
Expectations of senior residents	51–75%	26–50%
Resident pre-study survey		
Estimated past completion all levels	0–25%	0–25%
Junior residents	0–25%	0–25%
Senior residents	26–50%	26–50%
Expected capability all levels	51–75%	0–25%
Junior residents	26–50%	0–25%
Senior residents	76–100%	26–50%

### Initial resident perceptions and expectations

Thirteen residents (PGY-2–PGY-5) answered a survey evaluating their perceptions and expectations surrounding resident involvement in robotic cases. Of the residents who responded, three were PGY-2, four were PGY-3, three were PGY-4, and three were PGY-5. Ten residents had completed the robotic surgery curriculum provided by the residency program (consisting of online modules, live in person training with DaVinci sales representative, sitting at bedside/assisting, and simulation modules). The three residents who had not completed the training were PGY-2.

**Fig. 1:**
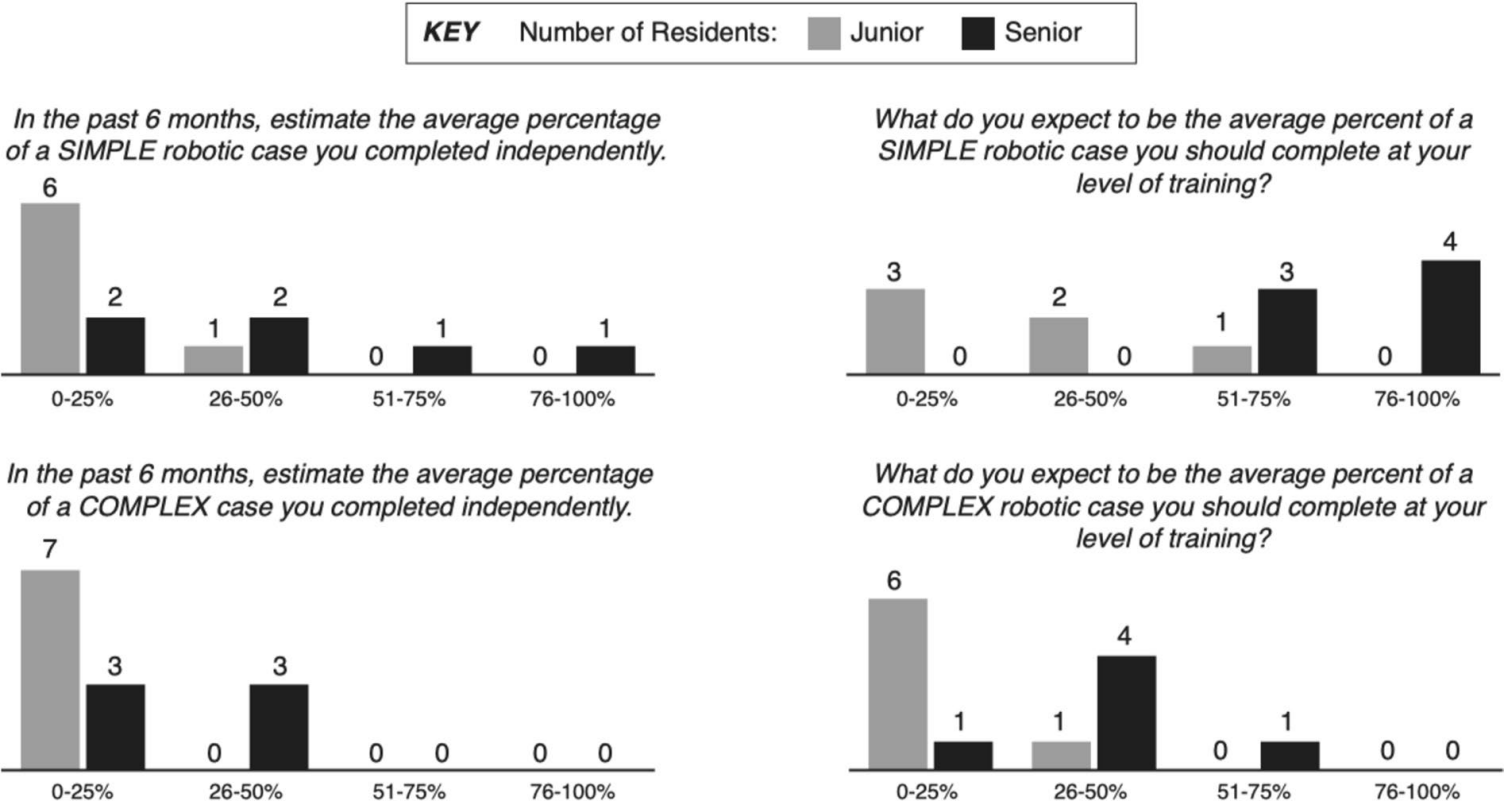
Pre-study survey results by seniority

Residents estimated that in the six months prior to beginning this study, they completed 0–25% of simple robotic cases (see Table 1 and Fig. 1), less than perceived by faculty. When examined by PGY, older residents did estimate that they had completed a greater proportion of simple cases than younger residents (*p* = 0.05).

Residents estimated that they completed 0–25% of complex robotic cases (see Table 1 and Fig. 1) on average (median). Only three residents (two PGY-5 residents and one PGY-4 resident) perceived that they completed more than 25% of a complex case but this was still significantly greater than perceptions of junior residents (*p* = 0.03).

Residents expected themselves to be capable of completing a higher proportion of simple robotic cases than they had been allowed in the previous six months. Residents on average (median) expected to complete 51–75% of a simple case (Table 1). All senior residents expected to complete at least 50% of a simple case (Fig. 1) which was significantly greater than junior level residents (*p* = 0.01) as well as greater than the median expectations of faculty. Both junior and senior residents perceived that they had been completing the appropriate proportion of complex cases.

### Post-case resident perceptions and expectations

Sixty-six post-case surveys were completed. Robotic cases performed included partial colectomy, abdominoperoneal resection (APR), low anterior resection (LAR), cholecystectomy, inguinal hernia repair, ventral hernia repair, and other. Other cases included resection of retroperitoneal mass, adrenalectomy, excision of pelvic cysts, ileoanal anastomosis/J-pouch, splenectomy, and parastomal hernia repair. The most frequent case performed and reported on was inguinal hernia repair (*N* = 28) followed by partial colectomy/APR (*N* = 10) and cholecystectomy (*N* = 10). Five LARs were completed and reported. Six ventral hernias were completed and reported on. Total other cases was six.

A large majority of cases were completed by senior residents (*N* = 58, 87.8%) compared to junior residents (*N* = 7, 10.6%). One survey was completed but the resident’s year was missing.

**Fig. 2:**
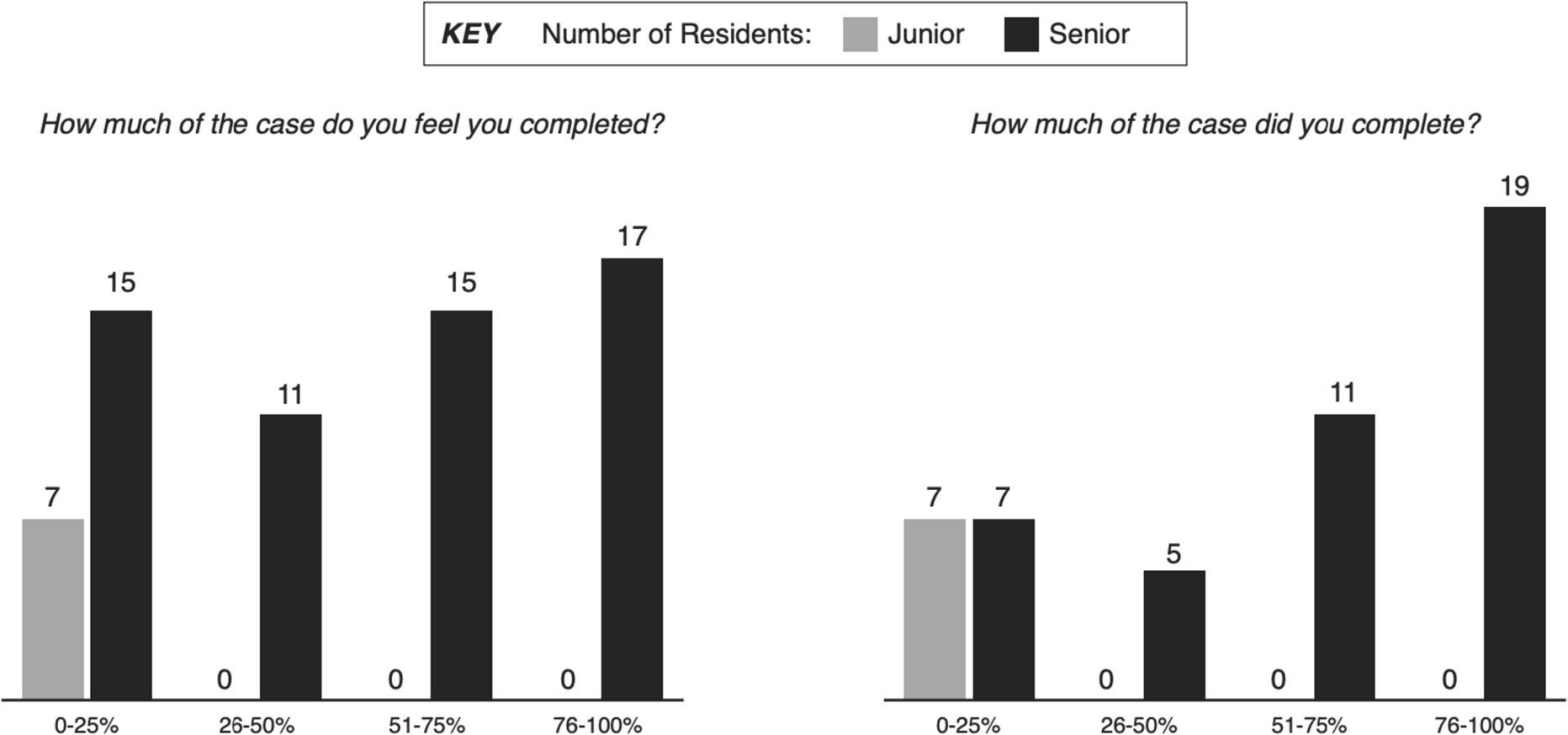
Post-case survey results by seniority

On average (median), residents estimated after any case that they had completed 26–50% of the case (see Table 2). Junior residents always estimated they had completed 0–25% of the case, regardless of the complexity, while senior resident responses ranged from 0 to 100% (see Fig. 2). Once examining their MyIntuitive report, they actually completed 51–75% of the case (median). However, there was no significant difference between what senior residents perceived the completed of an individual case compared to what they actually completed per the MyIntuitive report (*p* = 0.77). There was a significant difference between perceived completion by junior vs senior residents (*p* < 0.01) as well as actually completed (*p* < 0.01).

**Table 2:** Post-case survey results by seniority and case complexity

	Perception completed			Actually completed		
	All Cases	Simple	Complex	All Cases	Simple	Complex
All Levels	26–50%	51–75%	26–50%	51–75%	51–75%	26–50%
Junior Residents	0–25%	0–25%	0–25%	0–25%	0–25%	0–25%
Senior Residents	51–75%	51–75%	26–50%	51–75%	51–75%	26–50%

**Fig. 3:**
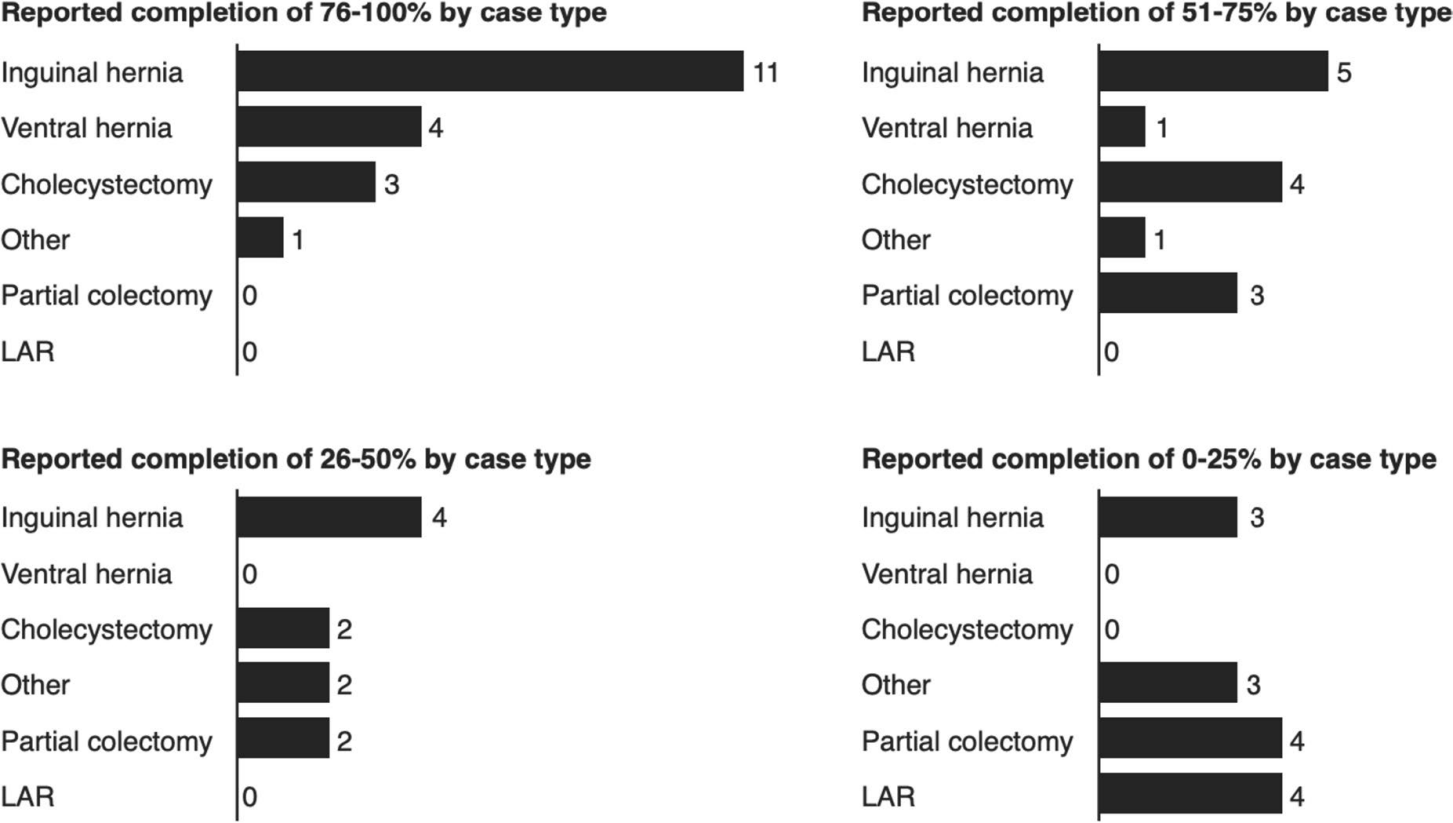
Most frequently completed cases by senior residents

Inguinal hernias were the most frequently performed and reported on cases. Over half of inguinal hernia cases reported on were completed at least 50% by a resident (Fig. 3). Other cases which residents completed a majority of included ventral hernia and cholecystectomy. Residents were more minimally involved in partial colectomy, APR, and other.

## Discussion

As robotic surgery has increased in popularity among general surgeons, our community hospital general surgery residency has sought to include residents in a greater proportion of robotic cases. As a result, we instituted a formalized training program which includes online educational modules, training with the DaVinci sales representative, skills drills on the robotic console, bedside assisting the surgeon using the robot and finally sitting at the console. This is consistent with residency programs around the country [5].

Few guidelines exist for continued growth beyond completion of these curriculae. At our institution, PGY-2 residents are encouraged to complete this curriculum and are increasingly assigned to robotic cases. In this study, we demonstrate that junior residents (PGY-2 and PGY-3) are gaining a small amount of exposure to live robotic cases and by PGY-4 and PGY-5 years, they are expected and allowed to complete increasing amounts of simple robotic cases (including inguinal hernia, cholecystectomy, and partial colectomy). Senior residents are utilizing skills learned on simple cases to assist in more complex cases, mostly LAR and ventral hernia repair and occasionally advanced hepatobiliary and surgical oncology cases. Utilizing this format improves upon previous resident curriculae which do not account for resident surgical experience in general and progression of autonomy throughout residency [5].

We observed that residents believe themselves to be, on the whole, capable of completing more robotic surgery than they perceive the are allowed to complete when asked to reflect on previous six months (Table 1). However, when console participation time was measured via the MyIntutive App and residents were asked to reflect on individual cases, residents are completing a larger proportion of cases than they realize—especially senior residents completing “simple” cases (Table 2). Despite initial faculty survey reflecting that residents are completing less than they would expect of robotic cases, it appears that after use of the MyIntuitive app, residents are participating about as much as faculty expected prior to beginning the study. Faculty expectations thereby are realistic and dictate resident involvement. One flaw of our study design was only asking about resident and faculty expectations in the pre-study period (in December or mid-academic year). Future investigation could be performed to see how resident and faculty expectations change over the course of the academic year.

When considering increasing faculty expectations and resident involvement in robotic cases, specific technical goals and expectations may satisfy both parties. Progression of autonomy using the robotic platform is similar to that of open or laparoscopic general surgery. Junior residents should begin to learn robotic skills while assisting or completing inguinal hernias, ventral hernias, and cholecystectomies. When creating a national curriculum for robotic training, consideration should be given to requiring graduating general surgery residents to be proficient in “simple cases” on the robotic platform. If an exam similar to Fundamentals of Laparoscopic Surgery (FLS) is created for robotic surgery, skills such as mobilizing the peritoneum, laying down a mesh into the inguinal space, suturing mesh in place, and closing the peritoneum could be assessed for passage. Breaking down cases into similar simple maneuvers has been demonstrated to be a safe and feasible method of teaching robotic thoracic surgery [10]. Senior residents may then expand upon these basic maneuvers to participate in more complex cases (colectomies, foregut, or hepatobiliary) which require further training in fellowship or in additional courses as a junior attending surgeon.

This study was limited by the small number of residents available for participation at our program (13 of 16 eligible participated). Further investigation into regional and residency type (academic vs community) trends should be completed by completing a similar study incorporating multiple programs. Another weakness of this study design is its lack of emphasis on junior residents. By inquiring about case participation time in quartiles, specific data are lost regarding how much junior residents are completing. Additionally, measuring participation during robotic cases by time operating may does not necessarily translate to the fraction of the case completed by the resident as certain steps may be more complex requiring more time and residents have varying levels of efficiency on the robotic platform. Further investigation into exactly how much time and which specific skills residents are working on as they are initiated to the robotic console is required. Using the results of this pilot study, we plan to design a study specifically focusing on progression of autonomy in robotic inguinal hernia repair by observing which steps of the case residents are completing and how long each of these steps takes. Finally, the MyIntutive App can only calculate the CPT easily when two consoles are used. Thus, many cases in which an attending and resident are switching on and off, one console was excluded (this would require each party logging in and out of their account during the case) and, thus, our residents are likely completing many more cases than this study reflects.

Much like laparoscopic surgery, robotic-assisted surgery is becoming a common tool of general surgeons around the world. As such, residents should be offered a seat at the console and allowed to develop skills so that they can enter practice with the ability to complete simple cases on the robotic platform.

## Figures and Tables

**Fig. 4:**
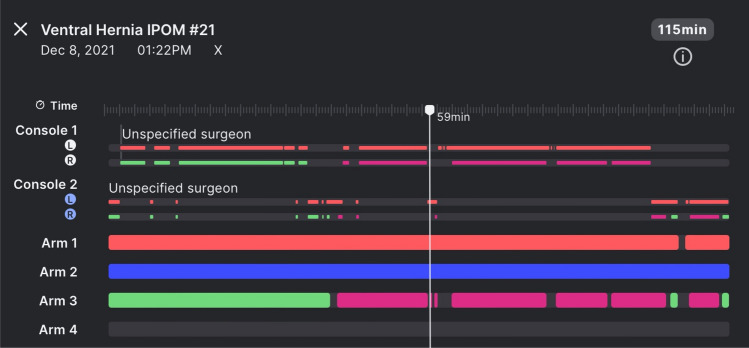
Sample screen capture of the My Intuitive App case timeline

## Data Availability

Not applicable.

## Appendix

### Appendix A: faculty survey

In the past 6 months, what do you estimate to be the average percent of a **simple*** robotic case you allow residents to independently complete (resident is controlling the robotic console but you may be giving verbal coaching)? Please circle an answer below.

0–25% 26–50% 51–75% 76–100%

In the past 6 months, what do you estimate to be the average percent of a **complex**** robotic case you allow residents to independently complete (resident is controlling the robotic console but you may be giving verbal coaching)? Please circle an answer below.

0–25% 26–50% 51–75% 76–100%

What do you expect to be the average percent of a **simple*** robotic case a resident **at the PGY-2 and PGY-3 level of training** should complete? Please circle an answer below.

0–25% 26–50% 51–75% 76–100%

What do you expect to be the average percent of a **simple*** robotic case a resident **at the PGY-4 and PGY-5 level of training** should complete? Please circle an answer below.

0–25% 26–50% 51–75% 76–100%

What do you expect to be the average percent of a **complex**** robotic case a resident **at the PGY-2 and PGY-3 level of training** should complete? Please circle an answer below.

0–25% 26–50% 51–75% 76–100%

What do you expect to be the average percent of a **complex**** robotic case a resident **at the PGY-4 and PGY-5 level of training** should complete? Please circle an answer below.

0–25% 26–50% 51–75% 76–100%

*Simple cases include inguinal hernia repair, cholecystectomy and partial colectomy.

**Complex cases include LAR, APR, Whipple, hepatectomy, abdominal wall reconstruction/midline hernia repair, and esophagectomy.

### Appendix B: resident pre-study survey

What is your PGY level?

Please circle one of the following.

PGY-2 PGY-3 PGY-4 PGY-5

Have you completed the robotic training curriculum created by Dr. Meister?

Please circle one of the following:

YesNo.

In the past 6 months, what do you estimate to be the average percent of a **simple*** robotic case you independently complete (you are controlling the console but may be receiving verbal coaching)? Please circle one of the following:

0–25% 26–50% 51–75% 76–100%

In the past 6 months, what do you estimate to be the average percent of a **complex**** robotic case you independently complete (you are controlling the console but may be receiving verbal coaching)? Please circle one of the following:

0–25% 26–50% 51–75% 76–100%

What do you expect to be the average percent of a **simple*** robotic case you (as a resident at your level of training) should complete? Please circle one of the following:

0–25% 26–50% 51–75% 76–100%

What do you expect to be the average percent of a **complex**** robotic case you (as a resident at your level of training) should complete? Please circle one of the following:

0–25% 26–50% 51–75% 76–100%

*Simple cases include inguinal hernia repair, cholecystectomy and partial colectomy.

**Complex cases include LAR, APR, Whipple, hepatectomy, abdominal wall reconstruction/midline hernia repair, and esophagectomy.

### Appendix C: resident post-case survey

What is your PGY level?

Please circle one of the following.

PGY-2 PGY-3 PGY-4 PGY-5

What case did you participate in while utilizing the DaVinci robotic surgery platform?

Please check one of the following.

o Inguinal hernia repair
o Ventral hernia repair
o Cholecystectomy
o Partial colectomy
o LAR
o APR
o Whipple
o Hepatectomy
o Abdominal wall reconstruction/ventral hernia repair
o Esophagectomy
o Other (if other, please specify _________)

#### How much of the case do you feel you completed?

What is your estimated console participation time (the percentage of time you were controlled the robot with attending coaching or instructing)? Please answer this before viewing your post-case report on the My Intuitive App. Please circle one of the following.

0–25% 26–50% 51–75% 76–100%

#### How much of the case did you complete?

Four hours after completing your case, please look at the case report in the My Intuitive App and calculate roughly what percentage you completed. For example, in Fig. 4, if you were on Console 1, you would have completed 75–100% of the case.

Please circle one of the following.

0–25% 26–50% 51–75% 76–100%
